# Improved lightweight YOLOv5 based on ShuffleNet and its application on traffic signs detection

**DOI:** 10.1371/journal.pone.0310269

**Published:** 2024-09-10

**Authors:** Liwei Liu, Lei Wang, Zhuang Ma

**Affiliations:** 1 Hebei Key Laboratory of Intelligent Data Information Processing and Control, Tangshan University, Tangshan City, Hebei Province, China; 2 Tangshan Key Laboratory of Intelligent Motion Control System, Tangshan University, Tangshan City, Hebei Province, China; University of Baghdad, IRAQ

## Abstract

Traffic signs detection is an important and challenging task in intelligent driving perception system. This paper proposes an improved lightweight traffic signs detection framework based on YOLOv5. Firstly, the YOLOv5’s backbone is replaced with ShuffleNet v2, which simplifies the calculation complexity and reduces the parameters of backbone network. Secondly, aiming at the problem of inconspicuous traffic sign characteristics in complex road environment, we use the CA attention mechanism in this paper to improve the saliency of the object. Finally, aiming at the large-scale difference between the traffic signs and the high proportion of small objects, we design the BCS-FPN to fuse multi-scale features and improve the representation ability of the small-scale objects. The TT-100K dataset is also analyzed and the dataset is collated. We test on the collated TT-100K dataset for the improved YOLOv5 in this paper. And the results show that compared with YOLOv5s, the mAP of our algorithm is equivalent to that of YOLOv5s, and the speed is improved by 20.8%. This paper also has carried on the experiment on embedded devices, experimental results show that our framework in computing power less embedded devices has a better effect.

## I. Introduction

With the rapid development of autonomous driving technology, the intelligent perception technology and vehicle communication technology of intelligent vehicles are also constantly updated and iterated [1–4]. Among them, road traffic signs detection [5, 6] is the key task of intelligent driving perception system. Road traffic signs of effective identification are the basis of the intelligent transportation system and unmanned technology, as well as the accuracy of the subsequent unmanned intelligent decision-making provides a convenient condition.

Recently, more and more traffic sign detection frameworks use CNNs, and the object detection algorithm based on CNNs has also achieved a lot of achievements. All the time, object detection has been the most fundamental and challenging branch of computer vision. The object detection frameworks based on CNNs are mainly divided into two categories. One is a two-stage object detection algorithm that pursues accuracy, and the other is a single-stage object detection algorithm that pursues speed. The difference between the two types of algorithms is based on whether the proposal region is further divided. Among them, the two-stage object detection algorithms will filter the proposal region and then match the prediction box. And the two-stage object detection algorithms mainly include R-CNN [7–9] series, Mask-RCNN [10] and Cascade R-CNN [11]. Domen Tabernik et al. [12] a CNN-based method to solve the whole process of target detection and recognition by training the model in an automatic end-to-end way. Y. Qian et al. [13] identified the problem of the single function of current deep learning models and proposed a unified neural network that can simultaneously detect drivable areas, lane lines, and traffic targets. The one-stage object detection algorithm will directly match the proposed region. And the single-stage object detection algorithms mainly include YOLO series [14–16] and SSD [17]. T. Suwattanapunkul et al. [18] used the YOLO series of algorithms to perform experiments on the Tsinghua-Tencent 100K (TT-100k), the Taiwan Traffic Signs (TWTS), and a hybrid dataset combining traffic scenes between TT100k and TWTS datasets. Y. Cao et al. [19] proposed a multi-scale small object detection structure to solve the problem of small-scale road traffic targets, and conducted experiments on the autonomous driving dataset BDD-100K.

The network frameworks of the existing algorithms are complex, the computational complexity is high, and the running memory occupied by the model is also large when it is deployed, which requires the device to have strong computing power support. In general, the computing power of vehicle processors is often poor, and the running memory is relatively small, so the above algorithm is not suitable for direct application in road traffic detection. For the problem of insufficient computing power of the device, some scholars have focused on lightweight detection networks. Andrew G. H et al. design MobileNet [20] network based on streamlined architecture, which uses depthwise separable convolution to build lightweight deep neural network. Subsequently, Andrew G. H et al. optimize MobileNet and propose MobileNet v2 [21] and MobileNet v3 [22] networks. Among them, the inverted residual with linear bottleneck structure is introduced in MobileNet v2, which has higher accuracy and smaller model than v1. MobileNet v3 updates the inverted residual structure of MobileNet v2, uses Neural Architecture Search (NAS) parameters, and finally redesigns the structure of the time-consuming layer. Huawei has also proposed a lightweight series network with similar performance to MobileNet, the GhostNet series [23–25]. The core idea of GhostNet is to generate feature maps that express intrinsic feature information with low-cost linear transformations. In addition, in a complex road environment, the above algorithms cannot effectively extract the object features, the detection effect of traffic signs with large-scale differences is not good, and the detection accuracy is not very high. For this problem, some scholars have paid attention to the attention mechanism which is widely used in the field of natural language processing. The essence of attention mechanism is to locate interesting information and suppress useless information. Hu J et al. propose a SENet [26] attention mechanism with low complexity, fewer parameters and less computation, including Squeeze part and Excitation part. Woo S et al. propose the CBAM [27] attention mechanism, emphasizing the features along the two main dimensions of the channel axis and the spatial axis.

Therefore, in view of the problems of large scale difference of traffic sign targets in complex road environment, complex detection model, and model deployment limited by equipment, this paper proposes a lightweight algorithm for traffic signs detection based on YOLOv5 [28] named SCB-YOLOv5. The main innovation and contribution are as follows:

1. To solve the problems that the complex model, the large number of model parameters, and the limited model by equipment during deployment, ShuffleNet v2 [29, 30] is used to replace the YOLOv5’s backbone network for extracting features, which greatly reduces the number of network parameters and improves the speed of network operation. And SimSPPF [31] is used to replace the SPPF structure, which improves the feature extraction ability of the backbone network.
2. Aiming at the problem that it is difficult to effectively extract object features in complex road environment, a lightweight CA [32] attention module is added to the backbone network, which enhances the saliency of the object at the cost of a small computational cost.
3. For the problem of large differences in target scales, the BCS-FPN is designed to replace the FPN+PAN structure of YOLOv5. SCCBL is used as the convolution module for the BCS-FPN to reduce the amount of model calculation while ensuring the accuracy. The C2f-SCConv structure is designed to further reduce the number of network parameters and improve the detection speed. Moreover, the multi-scale feature fusion mechanism is introduced to improve the network feature fusion ability.

The paper structure is as follows: Section II introduces the SCB-YOLOv5 and improves the details of each part, section III is the experimental results and analysis, and section IV is the conclusion.

## II. Methodology of the proposed approach

### 2.1 YOLOv5

**Fig 1:**
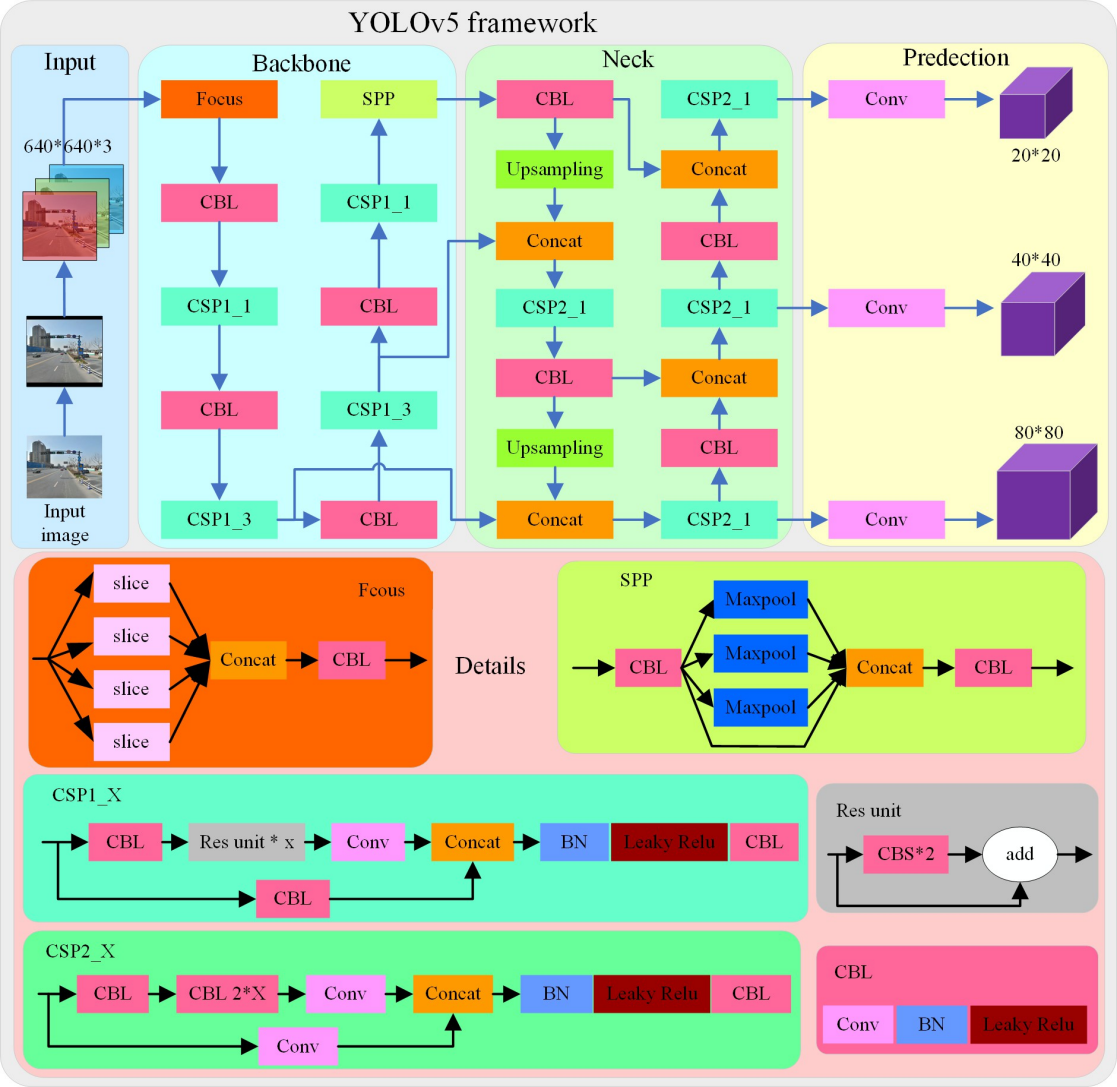
YOLOv5 algorithm structure.

YOLOv5 is the YOLO series algorithm used in most of the algorithms [33]. YOLOv5 is similar to YOLOv4 [34], but there are some differences. YOLOv5 algorithm than YOLOv4 backbone network part added Focus structure and CSP [35] structure. YOLOv5 is mainly divided into four parts, respectively input part, backbone network part, neck feature extraction part, and prediction part as shown in Fig 1.

The backbone network part mainly includes the Focus structure and the CSP structure. Focus mainly performs a slicing operation, which can reduce the size of the feature map by increasing the dimension of the feature map without losing any information. CSPNet takes the CSP structure for reference design train of thought, and joined the residual structure for effectively preventing the gradient from disappearing.

The Neck feature extraction network part adopts the FPN+PAN structure, which can effectively transfer semantic information and fuse multi-scale features. The Neck part also designs a CSP structure, which enhances the ability of the network to fuse multi-scale features while reducing the amount of calculation. At the prediction end, CIoU loss [36] is used as the bounding box regression loss, and NMS(non-maximum suppression) is used to screen the target box.

### 2.2 The improvements of the backbone network

We use the ShuffleNet v2 to replace the original YOLOv5’s backbone network for feature extraction. ShuffleNet series network is a kind of lightweight structure, its structure is clear and concise, and has verified on the multiple data sets its good generalization performance.

ShuffleNet v2 is the latest version of the ShuffleNet network family and proposes 2 principles for effective network architecture design, namely use direct metrics (such as speed) instead of indirect metrics (such as FLOPs) when designing networks and such metrics should be evaluated on the target platform. Based on these two principles, four principles for efficient network design are derived:

1. When the channels of the input feature matrix and the output feature matrix of the convolutional layer are equal, the MAC (memory access cost) is minimized, and FLOPs (floating-point operations) remain unchanged. For a convolutional layer with a 1×1 kernel, *hwc*_1_ is the memory consumption of the input feature matrix, *hwc*_2_ is the memory consumption of the output feature matrix, and 1×1×*c*_1_*c*_2_ is the memory consumption of the convolution kernel parameters, which can be obtained using the mean inequality since this condition is that FLOPs remain constant
$$\begin{array}{l} MAC=hw(c_{1}+c_{2})+c_{1}c_{2}\\ \;\geq 2hw\sqrt{c_{1}c_{2}}+c_{1}c_{2}\\ \;=2\sqrt{hw\mathrm{FLOPs}}+\frac{\mathrm{FLOPs}}{hw}, \end{array}$$ (1)
  where, FLOPs =*hwc*_1_*c*_2_, *h* and *w* are the height and width of the input and output feature matrices respectively, *c*_1_ and *c*_2_ are the number of channels of the input and output feature matrices respectively, and the condition for the above equation to take the equal sign is *c*_1_ = *c*_2_.
2. When the Group of GConv(Group Convolution) increases, the MAC will also increase (keeping FLOPs constant). For a 1×1 convolutional layer in Group Conv, *hwc*_1_ is the memory consumption of the input feature matrix, *hwc*_2_ is the memory consumption of the output feature matrix, and $1\times 1\times (c_{1}/g)\times (c_{2}/g)\times g$ is the memory consumption of the convolution kernel parameters. Among them, *g* is the number of Groups. Thus, it can be obtained
$$\begin{array}{l} MAC=hw(c_{1}+c_{2})+\frac{c_{1}c_{2}}{g}\\ \;=hwc_{1}+\frac{\mathrm{FLOPs}\times g}{c_{1}}+\frac{\mathrm{FLOPs}}{hw}. \end{array}$$ (2)
  Among them, FLOPs =*hwc*_1_*c*_2_/*g*, when the fixed FLOPs are unchanged, the increase of *g* will cause the increase of MAC.
3. When the network design is more fragmented, the processing speed is slower. There are many branches in networks such as Inception and SPP block, and the degree of fragmentation is also the degree of branches, which can be in parallel or series. Although the fragmented structure can improve the accuracy, it will decrease the efficiency of the model. The fragmented structure is also not suitable for running on GPU devices with strong parallel capabilities, and the start and synchronization of convolution kernels are also involved in the case of many branches. So the more fragmented the network design, the slower it will be.
4. Element-wise overhead also slows things down. Element-wise operations include activation functions, Element addition (residual structure), etc., and bias in convolutions. The commonality of Element-wise operations is that FLOPs are small, but MAC is large. Moreover, the DW Conv(Depthwise Convolution) can also be seen as an Element-wise operation. In practice, Element-wise operations are more time-consuming than expected.

**Fig 2:**
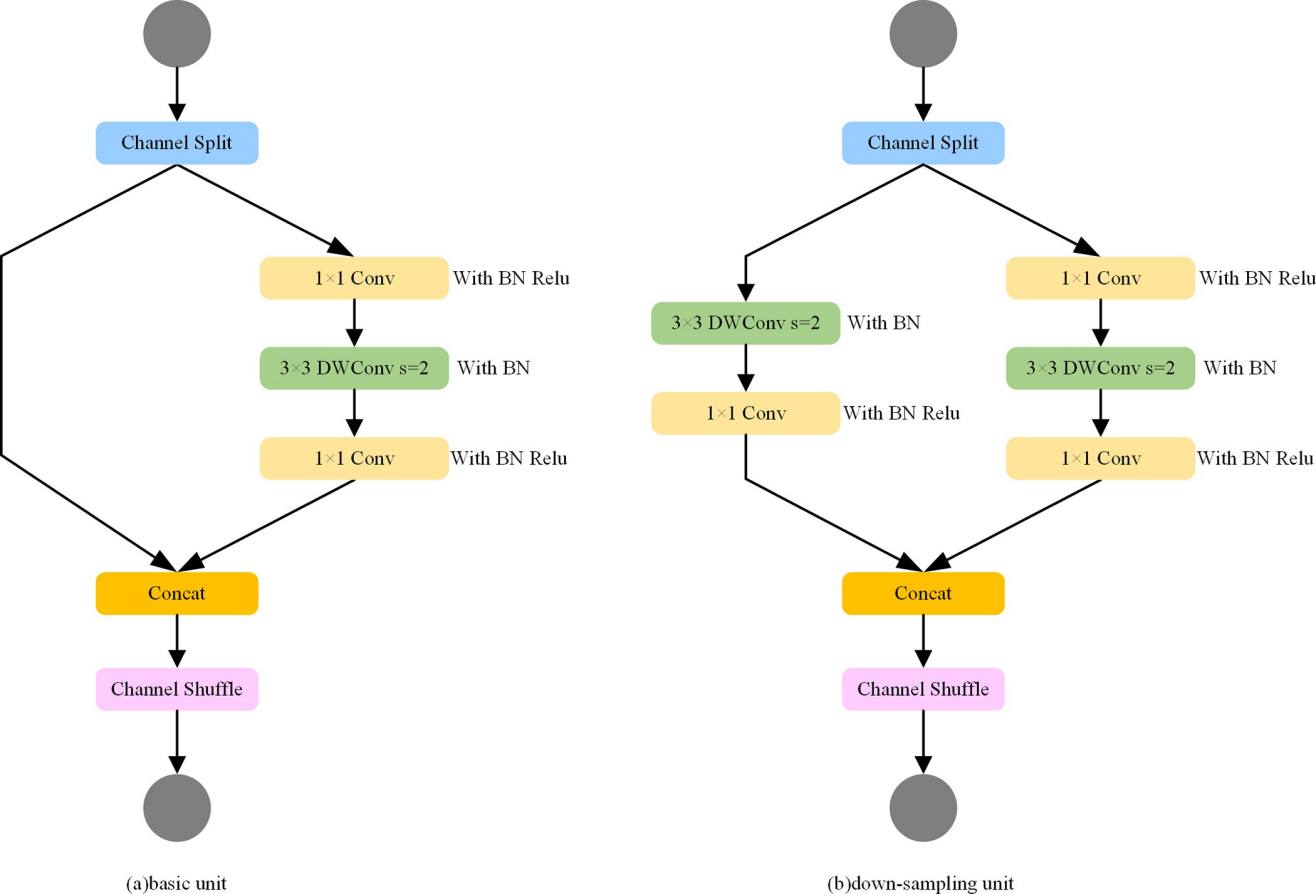
The ShuffleNet v2 network structure diagram. The Fig 2(A) on the left is the basic feature extraction unit with residual structure, and the Fig 2(B) on the right is the down-sampling unit.

According to the above four design criteria, the ShuffleNet v2 can be designed, and the structure is shown in Fig 2. For the basic unit of the ShuffleNet v2 network shown in Fig 2(A), the channel of its feature input matrix is split into two branches. Firstly, aiming at the principle of simplifying the complexity of the network, the degree of fragmentation is reduced in the design of the subsequent network. No operation is added in the left branch, and the number of channels of the three Conv inputs and outputs in the right branch is the same, which also meets the principle of the equal number of input and output channels. After Conv, the two branches are concatenated using Concat, which also makes the number of front and back channels consistent for the whole unit. Channel reorganization is then performed at the end of the unit. Add operations are no longer performed in the whole basic unit, and the ReLU activation function and DW Conv only exist in one branch, reducing Element-wise operations as much as possible. For the ShuffleNet v2 down-sampling unit shown in Fig 2(B), the operation of channel splitting is canceled, and finally, the channels of the output feature matrix are doubled after Concat. The 3×3 average pooling of one of the branches is turned into a 3×3 DW Conv, which can be regarded as a DW Conv with a weight of one-ninth, which can increase more possibilities, and x finally adds a 1×1 convolution. In particular, in both units of ShuffleNet v2, DW Conv is followed only by the BN layer, cancelating the ReLU layer.

**Fig 3:**
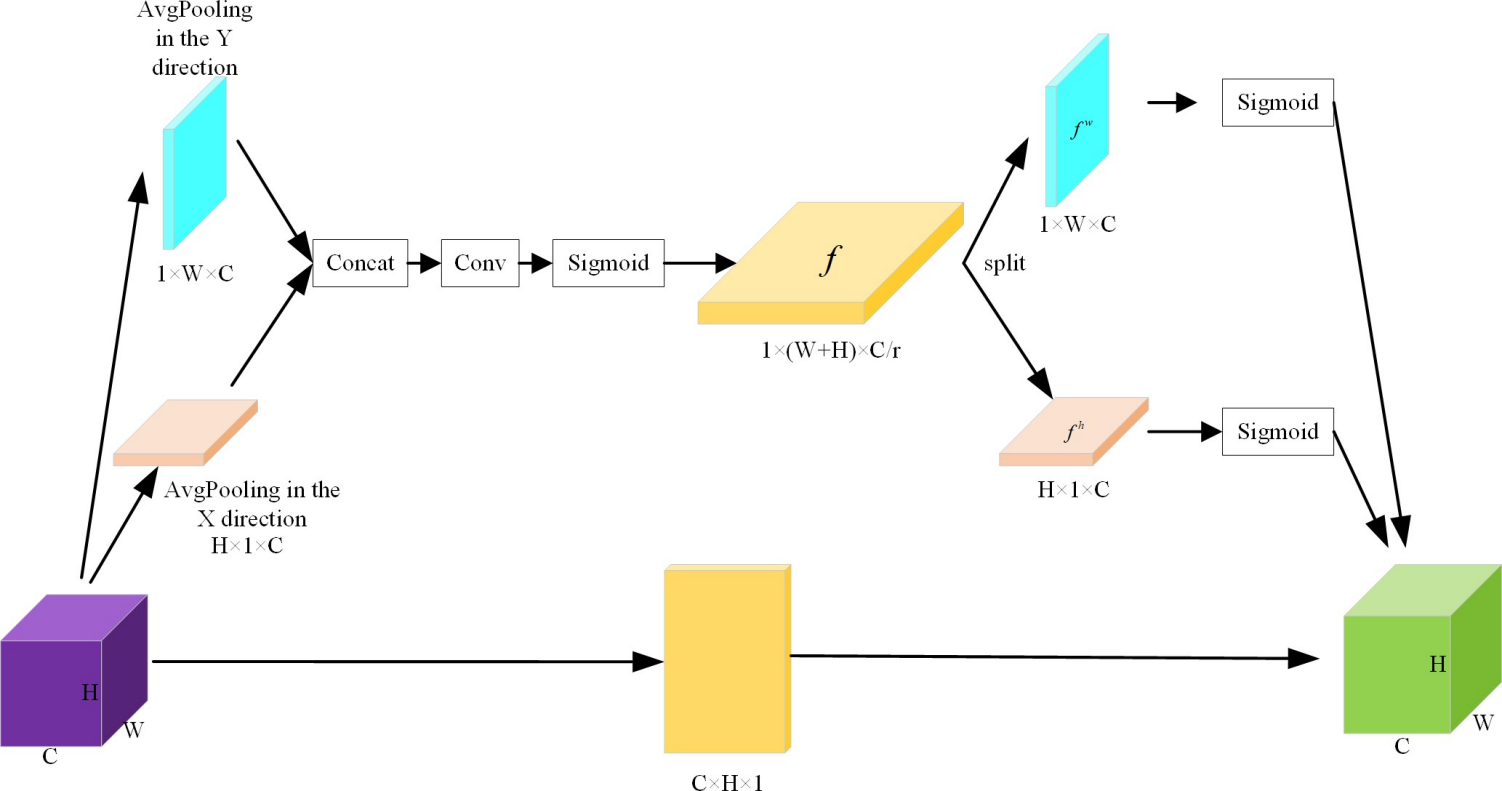
Structure of CA attention mechanism.

In this paper, after extracting features in the backbone network, to strengthen the saliency ability of feature expression, the CA attention mechanism is added to the back of the backbone network. CA attention mechanism is a lightweight attention mechanism that can effectively enhance the expression ability of network learning features, and its implementation process is shown in Fig 3.

The main implementation process of the CA attention mechanism is that location information is embedded in channel attention to encode channel relationships and long-term dependencies through accurate location information. In the left part of Fig 3, to capture the attention and encode the position information in the width and height of the image, the input feature map is first divided into two directions of width and height, and the global average pooling is performed respectively, so that the feature maps in the width and height directions are obtained, as shown in the following equation:

$$\begin{array}{l} z_{c}^{h}=\frac{1}{W}\sum_{0\leq i\leq W}x_{c}(h,i),\\ z_{c}^{w}=\frac{1}{H}\sum_{0\leq j\leq H}x_{c}(j,w). \end{array}$$ (3)

Among them, $z_{c}^{h}$ is the output of the *c*−*th* channel with height *h* and $z_{c}^{w}$ is the output of the *c*−*th* channel with width *w*. These two transformations aggregate features along two spatial directions, respectively, resulting in a pair of direction-aware feature maps. Then, the two feature maps are concatenated together by the Concat operation and fed into the shared convolution module to reduce its dimension to the original *C*/*r*, as shown in the following equation:

$$f=\delta (F_{1}([z^{h},z^{w}])).$$ (4)

Among them, [∙,∙] is the Concat operation along the spatial dimension, *δ* is the nonlinear activation function, and *f* is the intermediate feature map that encodes the spatial information both horizontally and vertically. *f* is then decomposed into two separate tensors *f*^*h*^∈*R*^*C*/*r*×*H*^ and *f*^*w*^∈*R*^*C*/*r*×*W*^ along the spatial dimension. Then two other 1×1 convolution transformations are used to transform *f*^*h*^ and *f*^*w*^ into tensors with the same number of channels, resulting in:

$$\begin{array}{l} g^{h}=\sigma (F_{h}(f^{h})),\\ g^{w}=\sigma (F_{w}(f^{w})), \end{array}$$ (5)

where *σ* is the Sigmoid activation function. Finally, the weight *g*^*h*^ in the height direction and the weight *g*^*w*^ in the width direction of the obtained input feature map are jointly weighted on the original feature map to obtain the feature map with weights in the height direction and width direction, as shown in the following equation:

$$y_{c}(i,j)=x_{c}(i,j)\times g_{c}^{h}(i)\times g_{c}^{w}(j).$$ (6)

**Fig 4:**
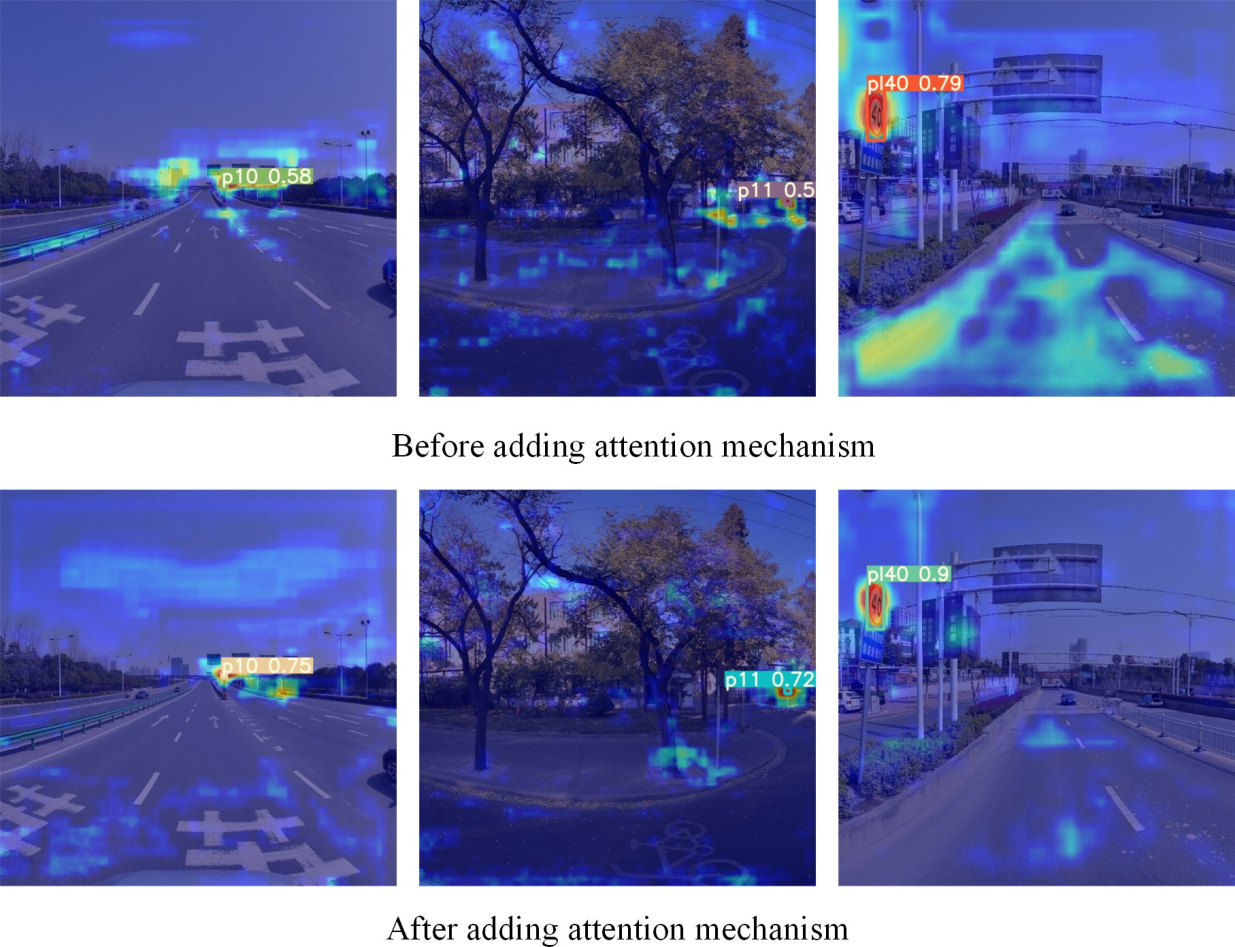
Comparison of thermodynamic diagrams before and after adding the CA attention mechanism.

In this paper, the CA attention mechanism is added to the tail of the backbone network. Fig 4 shows the comparison before and after adding the CA attention mechanism. In Fig 4, red regions indicate regions with high saliency for a certain object, and darker colors indicate higher saliency.

**Fig 5:**
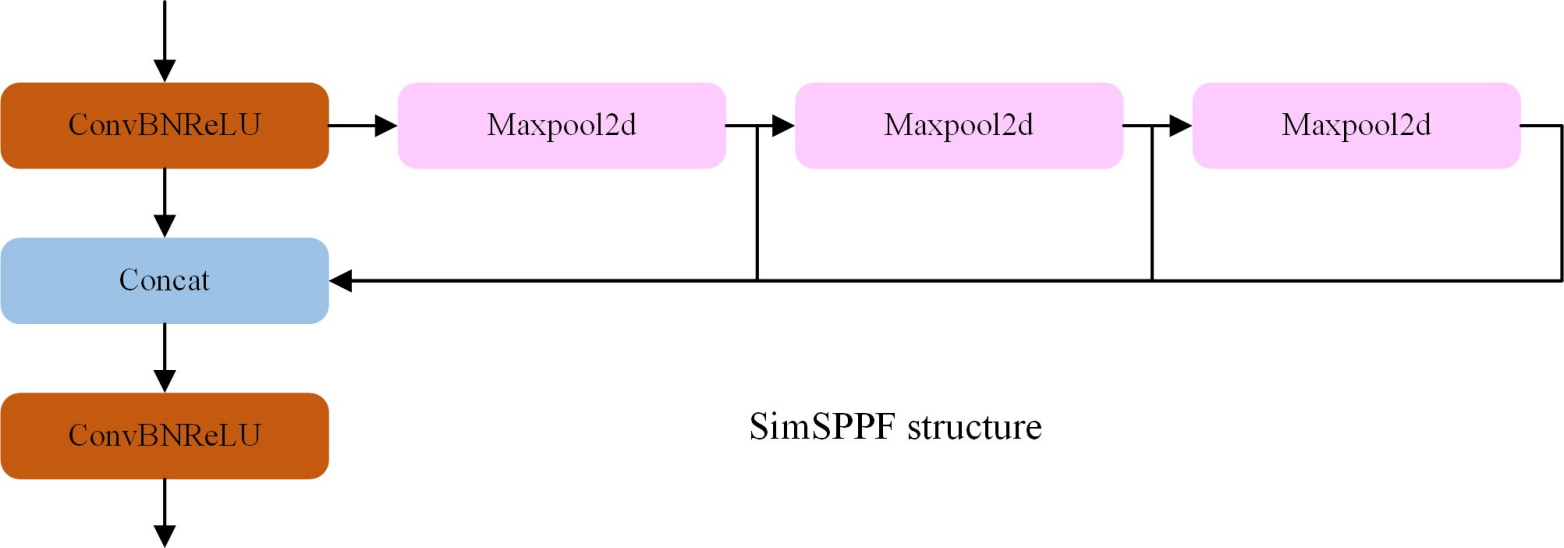
SimSPPF structure diagram.

In this paper, the SPP structure is improved by using SimSPPF for replacement and replacing the Leaky ReLU activation function with the ReLU activation function, and the specific structure is shown in Fig 5.

**Fig 6:**
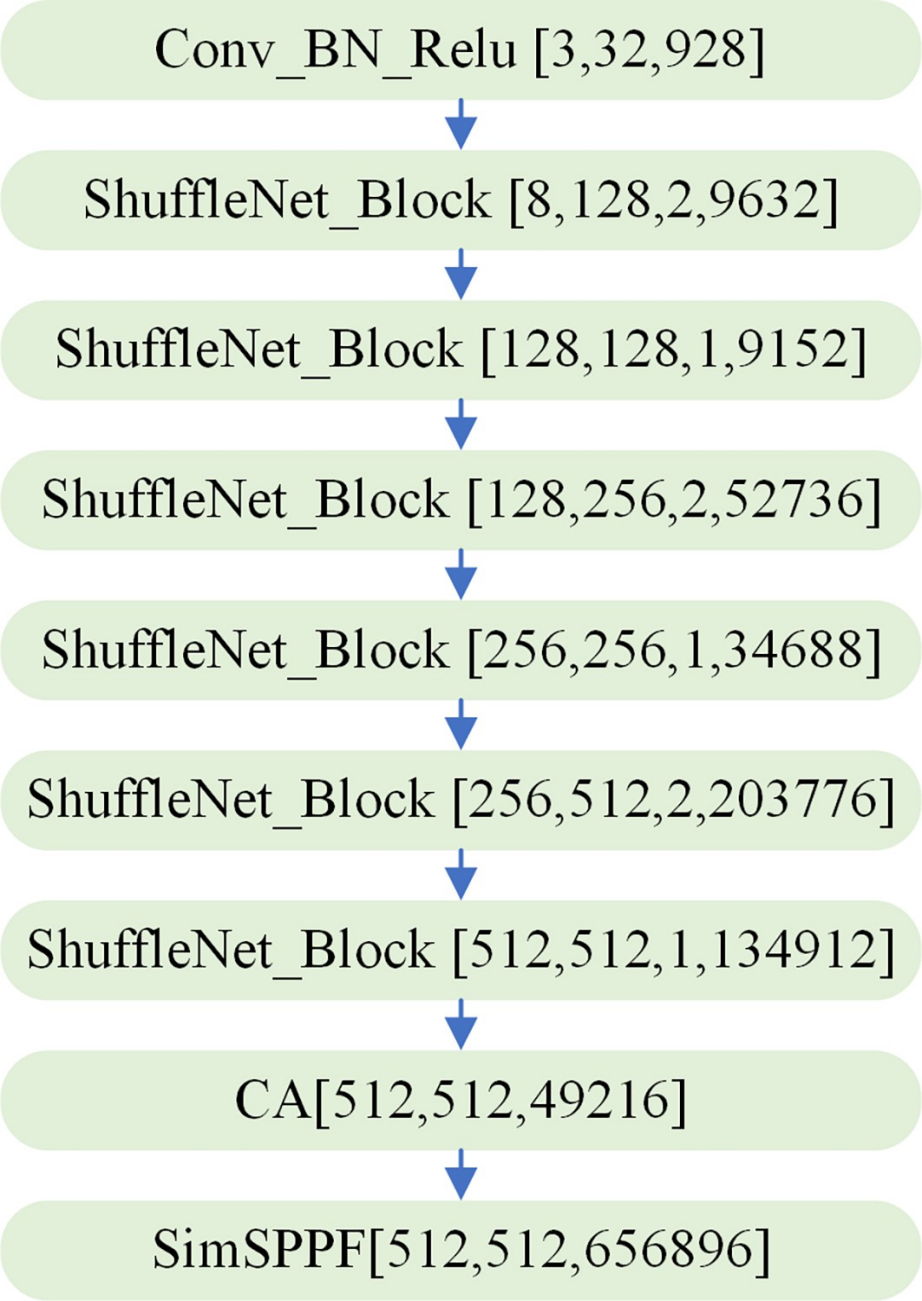
The parameters of the backbone.

Finally, the backbone network parameters used in this paper are shown in Fig 6.

In Fig 6, the parameters in parentheses after Conv_BN_Relu and SimSPPF represent the number of input channels, the number of output channels, and the number of parameters, respectively. The parameters in parentheses after ShuffleNet_Block represent the number of input channels, the number of output channels, the module category, and the number of parameters, respectively, where, when the module category is 1, it is the base module of ShuffleNet v2, and when the module category is 2, it is the down-sampling module of ShuffleNet v2. CA modules after the parameters in parentheses, respectively for the number of input channel, output channel number, and reference number. After calculation, the improved backbone network parameter quantity is 0.49M, and the calculation amount is 1.4GFLOPs, while the backbone network parameter quantity of YOLOv5s is 3.82M, and the calculation amount is 9.6GFLOPs. Thus, after replacement of backbone network, the model parameter was reduced by 69.1%, the amount of calculation was reduced by 78.1%.

### 2.3 The improvements in feature fusion networks

**Fig 7:**
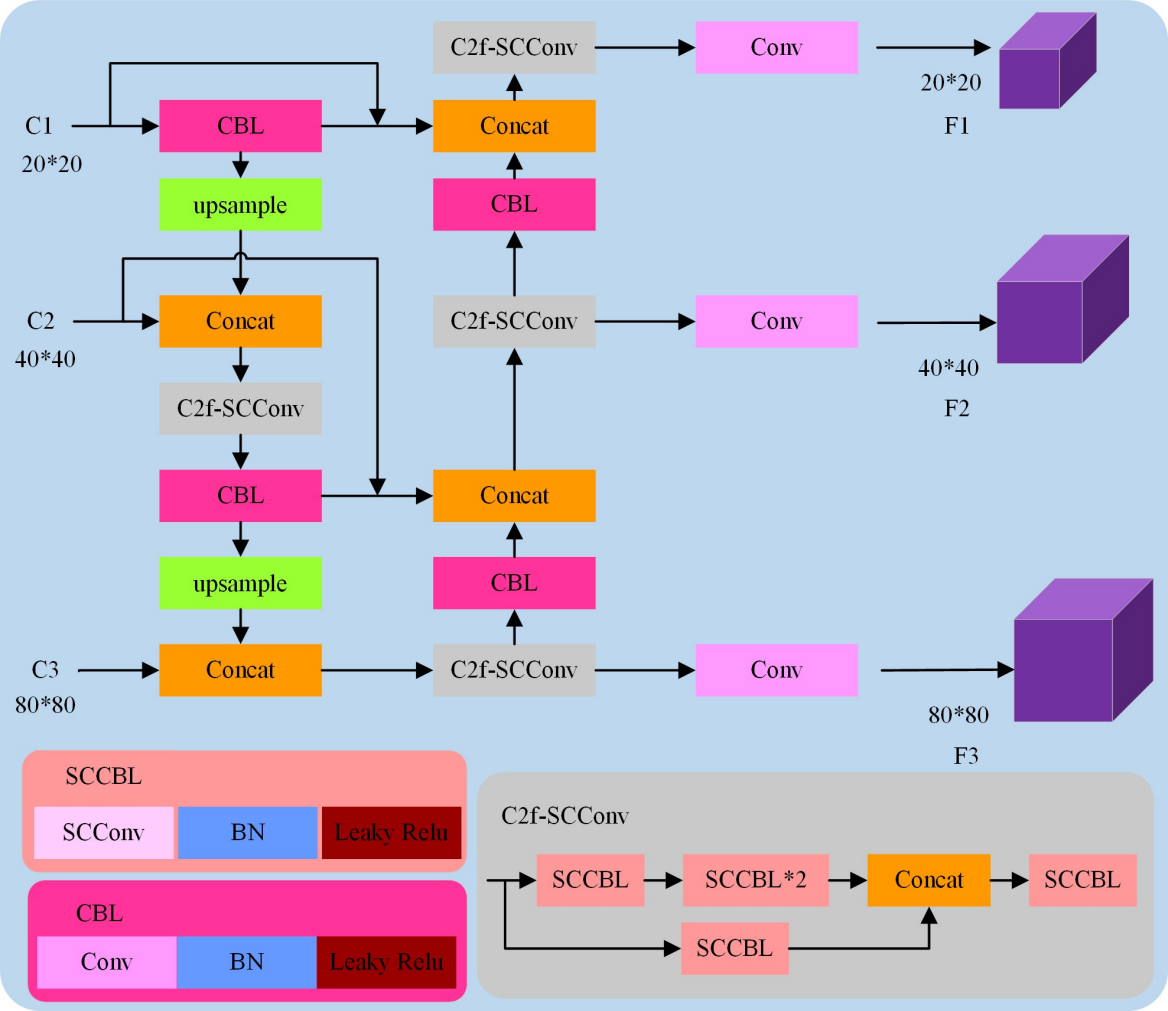
Diagram of the BCS-FPN structure.

The neck of YOLOv5 uses the FPN+PAN structure. Because this method is a top-down and then bottom-up feature fusion mechanism, it can only fuse the feature map features of adjacent scales, and it is difficult to effectively fuse the cross-scale features. To explore a feature fusion network that can fully fuse multi-scale feature maps, so that the three outputs of the network can fully fuse the features of different levels, and can only increase a small amount of parameters to ensure the detection speed of the network, this paper designs a lightweight cross-scale feature fusion mechanism named BCS-FPN, which is the FPN [37] with BiFPN [38] and C2f-SCConv, as shown in Fig 7.

The improvement of BCS-FPN compared with the FPN+PAN structure in YOLOv5 is that a lightweight module is designed for the feature fusion network to reduce redundant calculations and a multi-scale feature fusion mechanism is introduced to enhance the feature fusion ability. In Fig 7, C2f-SCConv is the designed lightweight module, among which, the SCCBL module is an efficient convolution module and uses SCConv [39] as the basic unit of convolution. SCConv uses characteristics between space and channel redundancy to compress the CNN, which reduces the representative characteristics of redundant computation and is easy to learn. The implementation process of BCS-FPN is as follows: Firstly, the feature map output by the C1 layer is connected to the prediction end of the F1 layer, and the feature map output by the C2 layer is connected to the prediction end of the F2 layer, so that an edge from the original input node to the output node is added, which can fuse as many target features as possible under the premise of increasing a small amount of calculation. Secondly, the convolution module is replaced by the SCCBL module to further reduce the amount of calculation while ensuring accuracy. Finally, the C2f-SCConv structure was designed for the feature fusion network to further reduce the number of parameters and improve the detection speed.

**Fig 8:**
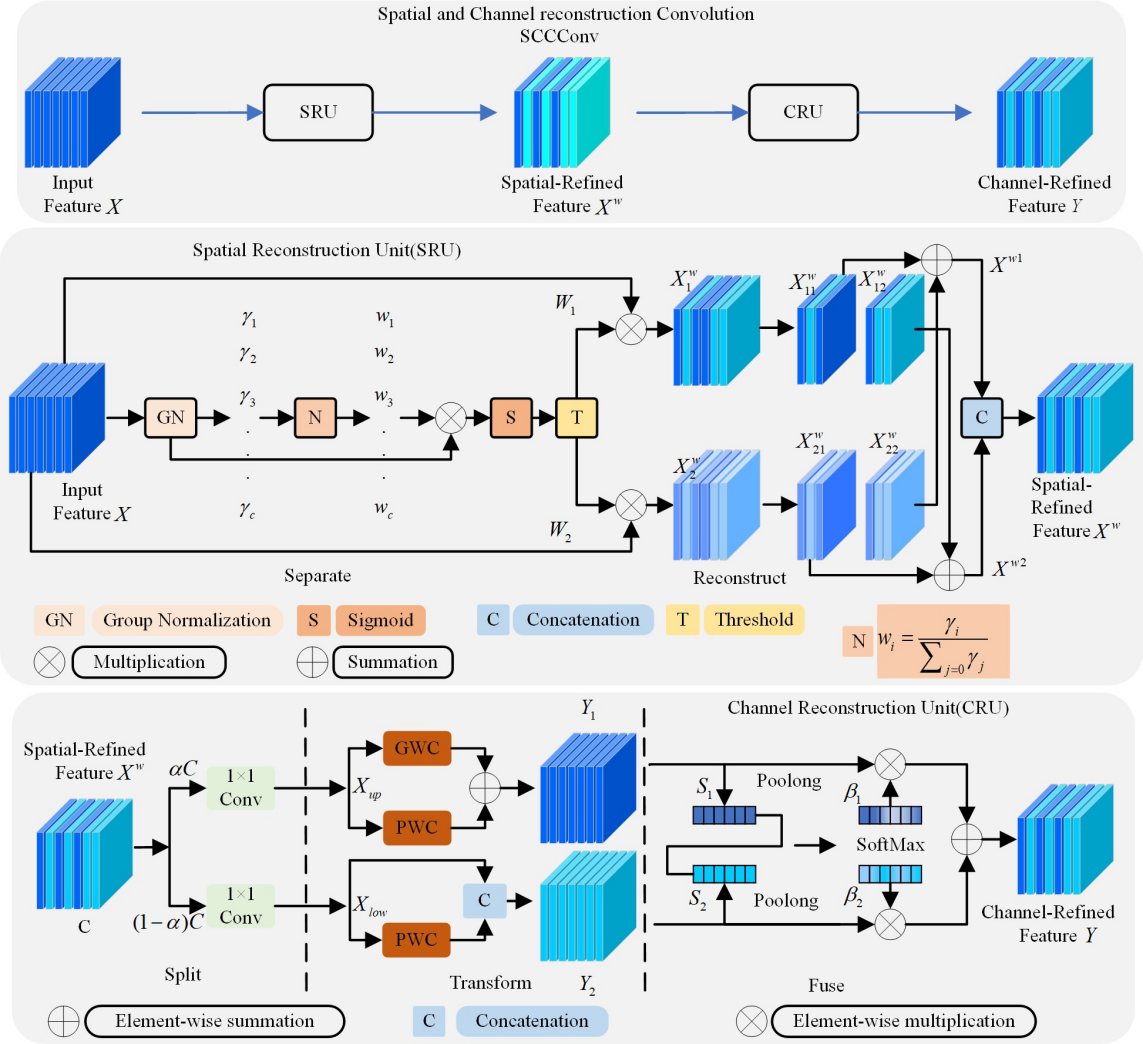
Diagram of the SCConv structure.

SCConv structure is shown in Fig 8. In the first part of Fig 8, SCConv(Spatial and Channel reconstruction Convolution) consists of a spatial reconstruction unit (SRU) and a channel reconstruction unit (CRU). SRU utilizes a separate-and-reconstruct method to suppress the spatial redundancy while CRU uses a split-transform-and-fuse strategy to diminish the channel redundancy. Concretely, for the intermediate input feature *X* in the bottleneck residual block, the spatial refinement feature X^w^ is firstly obtained by SRU operation, and then the channel refinement feature *Y* is obtained by CRU operation. Exploiting the spatial and channel redundancy between features in the SCConv module, it can be seamlessly integrated into any CNN architecture to reduce redundancy between intermediate feature maps and improve the feature representation of CNNs.

The role of SRU is to exploit the redundant features of the space, as shown in the SRU structure in the middle part of Fig 8, with the operation of separation reconstruction. The purpose of separation operation is characteristic of the information rich content for less characteristic figure and space separation. The scaling factor in the Group Normalization(GN) layer is then used to evaluate the information content of the different feature maps. And we leverage trainable parameters in the GN layer to measure the variance of spatial pixels for each batch and channel. Then the weight values of the feature map are mapped to [0,1] by sigmoid function. Finally, the input features are multiplied by the weight values *W*_1_ and *W*_2_ respectively to obtain two weighted features $X_{1}^{w}$ and $X_{2}^{w}$, and the Spatial-Refined features are obtained by adding them by the Reconstruct operation. CRU’s role is to use the channel characteristic of redundancy, the CRU structure as shown in the last part of Fig 8, the split-transform-fusion strategy, further reducing space refined characteristic figure X^w^ along the channel dimension redundancy. The Split operation splits the channel of the spatially refined feature into two parts, one with *α*∙*C* channels and the other with (1−*α*)∙*C* channels, where 0≤*α*≤1 is a split ratio. Then 1×1 convolution is used to compress the channels of the feature map to improve efficiency. In Transform operation, efficient convolution operations (GWC and PWC) are used instead of standard convolution to extract high-level representative information and reduce the computational cost. In Fuse operation, global average Pooling is first applied to collect global spatial information. The global channel descriptors *S*_1_ and *S*_2_ of the upper and lower parts are then stacked together and the channel attention operation is used to generate the feature importance vector *β*_1_ and *β*_2_. Finally, *Y*_1_ and *Y*_2_ are merged in a channel manner under the guidance of the feature importance vector. In addition, rich representative features can be extracted by CRU, and redundant features can be handled through low-cost operations and feature reuse while lightweight convolution operations.

After calculation, the parameter amount of BCS-FPN is 1.9M, and the calculation amount is 3.6 GFLOPs. The parameter amount of YOLOv5s neck part is 2.45M, and the calculation amount is 4.6 GFLOPs. It can be seen that after replacing the FPN+PAN of the YOLOv5s with BCS-FPN, the parameter amount is reduced by 22.4%. The amount of calculation is reduced by 21.7%.

### 2.4 SCB-YOLOv5

**Fig 9:**
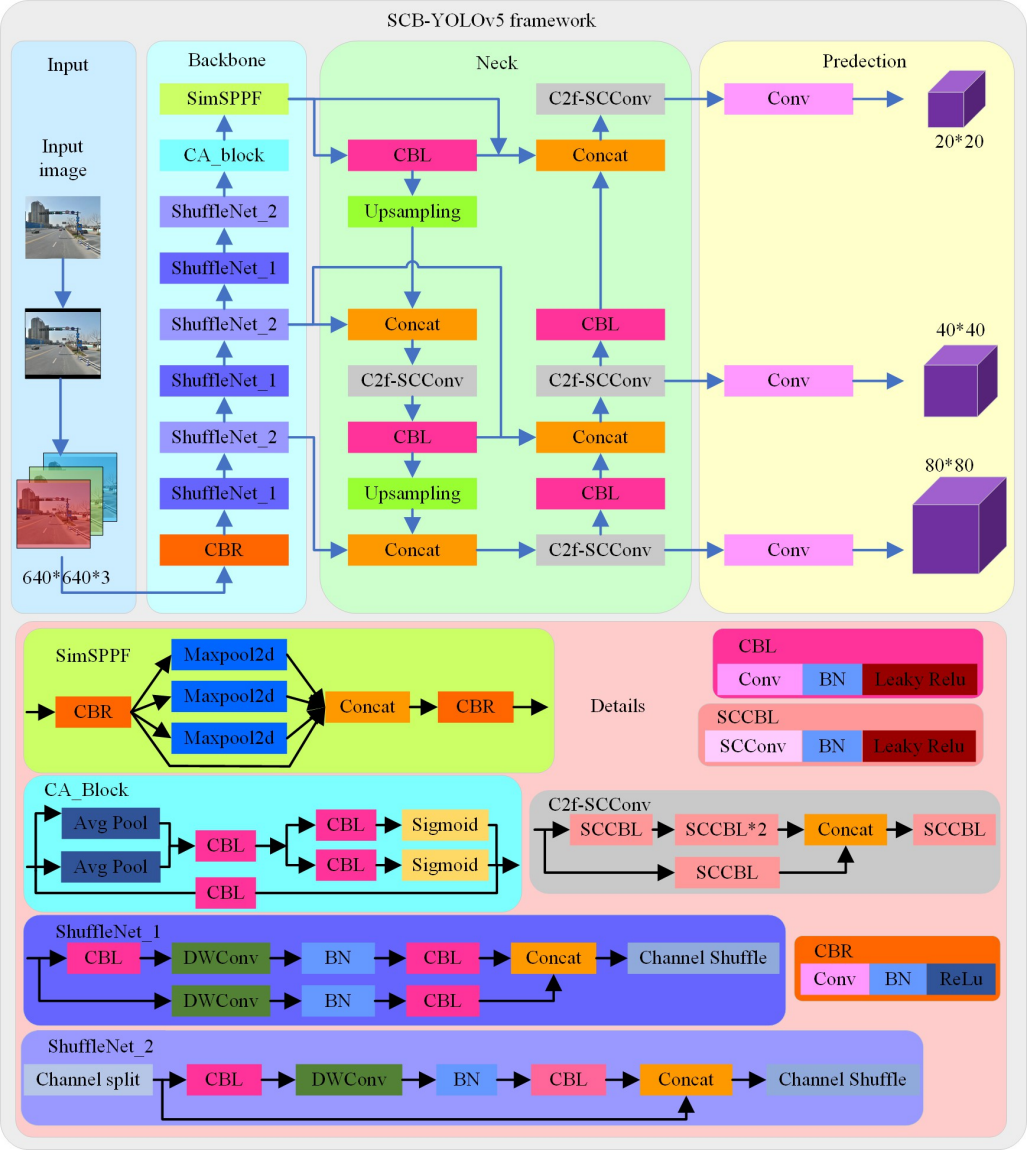
Diagram of the SCB-YOLOv5 structure.

The lightweight traffic sign detection framework based on YOLOv5 named SCB-YOLOv5 is shown in Fig 9. The SCB-YOLOv5 is that the YOLOv5 with ShuffleNet, CA attention mechanism, and BCS-FPN.

As shown in Fig 9, SCB-YOLOv5 is mainly divided into four parts, namely input, backbone, neck and prediction. First, images are fed to the network from the input, and after being scaled and padded, 640*640*3 images are fed to backbone. Secondly, the ShuffleNet v2 network is used for feature extraction, the CA attention mechanism is used to enhance the saliency of the target, and the SimSPPF module is used to handle the distortion of the image to enhance the feature extraction ability. Then, multi-scale feature fusion is performed through the BCS-FPN network. Finally, inference of prediction boxes is performed at three scales. The process of the detection framework in this paper is as follows:

1. The TT-100K data set is analyzed and divided into the training set, validation set, and test set.
2. The optimal weights of the model are obtained by training the network with the divided training set and validation set.
3. The obtained model training weights are used to perform a validation test on the test set.

## III. Experimental results and comparative analysis

In the experiments of this paper, the SCB-YOLOv5 model is comprehensively analyzed on the TT-100K dataset. The experiment is carried out under the Pytorch framework. The operating system of the server is Linux Ubuntu 18.04, the CPU model is Intel(R) Xeon(R) Gold 6248R, the CPU frequency is 3.00GH, and the memory of the server was 128GB DDR4. The GPU is RTX A6000, with memory of 48 GB.

### 3.1 Analysis of the TT-100K dataset

**Fig 10:**
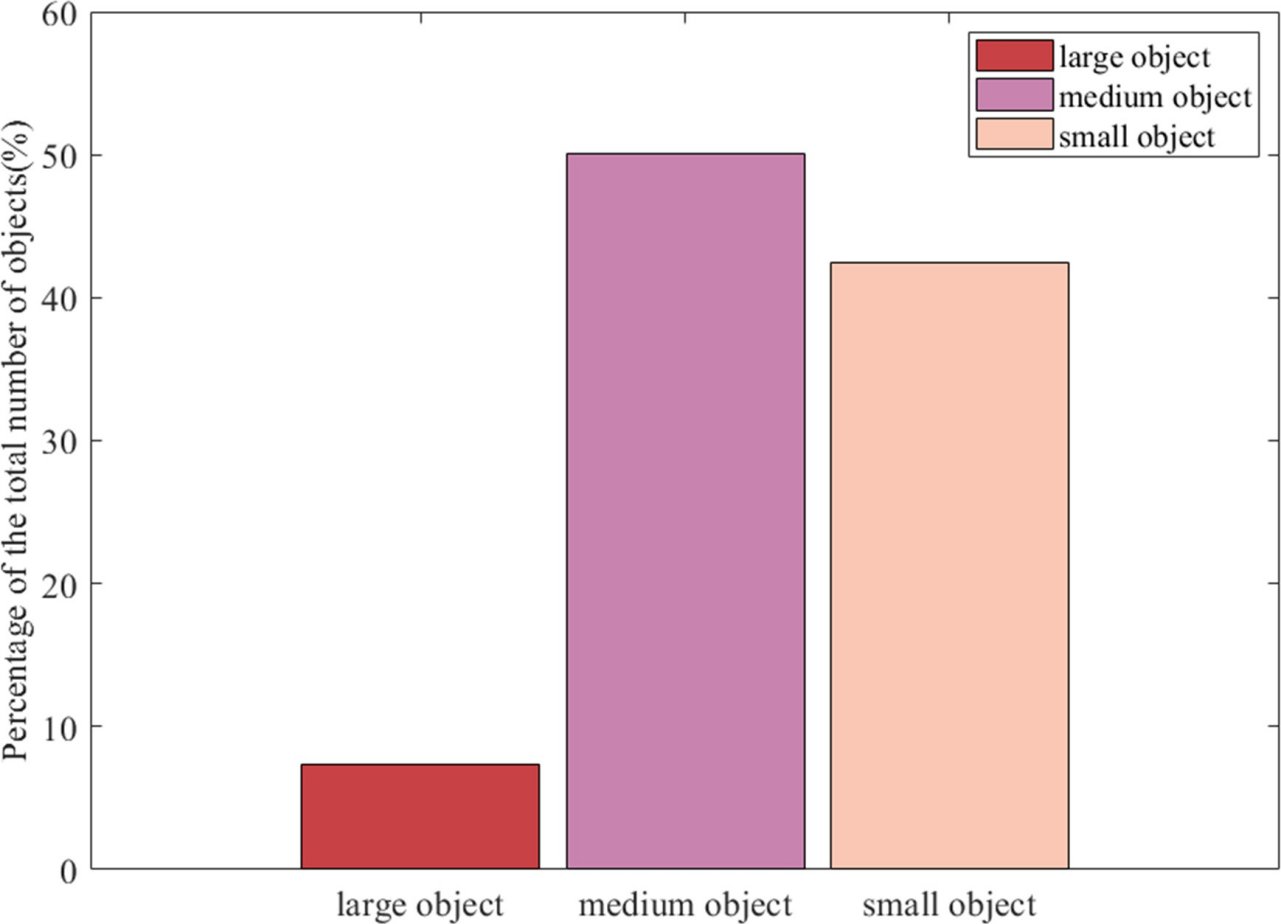
Scale statistical plot of the TT-100K dataset.

In this section, we first present an analysis of the TT-100K dataset. TT-100K dataset is a commonly used traffic sign dataset jointly produced by Tsinghua University and Tencent. Among them, the training set contains 6105 images and the test set contains 3071 images, and the data set contains 232 kinds of traffic signs in total. In this paper, the number of targets and the scale of targets in the TT-100K dataset are first counted, and the statistical results are shown in Fig 10. We then classify the objects into three categories based on their pixel size. The small-scale objects, whose pixel size is less than 32×32. The mesoscale objects, whose pixel size is between 32×32 and 96×96. Large scale objects, whose pixel size is greater than 96×96.

Because the data set of some categories of quantity is less, easy to cause in the process of training network owe fitting. So to ensure the validity of the model, this paper only chooses a target quantity is more than 100 categories to continue training. Finally, the categories of the filtered dataset and their numbers are shown in Table 1.

**Table 1:** After screening of TT-100K data set object and quantity.

Labels	Numbers	Labels	Numbers	Labels	Numbers
pne	2091	pn	2963	pl40	1356
p5	393	i2r	417	pl50	1027
pl60	820	i5	1582	w57	393
pl80	865	pl12	181	pl120	296
pr40	200	pl5	484	w59	196
pl30	596	i4	733	pm20	156
ip	340	pl100	664	p23	281
pm30	107	pg	154	p26	812
w55	175	p10	358	p11	1535
p13	353	p19	122	i4l	330
ph4.5	186	pl20	158	p3	169
pl70	149	il80	294		

### 3.2 Network model training

Before training the model, the ratio of the training set to the validation set was divided into 7:3. The image input model, carries on the pretreatment, and adjust the picture size is 640 x 640. The training method uses SGD with a momentum parameter size of 0.937, an initial learning rate of 0.01, and 16 images for each batch. All models are trained for 300 epochs according to these parameters.

### 3.3 Experimental results and analysis

To objectively evaluate the advantages of the SCB-YOLOv5 algorithm proposed in this paper, Precision, Recall, average precision mAP@50, and average inference time are selected as evaluation indicators, and the calculation formula is as follows.

$$precision=\frac{TP}{TP+FP},$$ (7)

$$recall=\frac{TP}{TP+FN},$$ (8)

$$\begin{array}{c} AP=\int_{0}^{1}P_{\left(R\right)}dR,\\ mAP=\frac{\sum_{i=1}^{N}AP_{i}}{N}, \end{array}$$ (9)

where, *TP* is the number of correct detections, *FP* is the number of false detections, is the number of missed detections, *FN* is the integral of the Precision-Recall curve, *AP* is the number of detection categories, and the average inference time in this paper is the average time calculated after 500 images which are selected for testing.

The SCB-YOLOv5 traffic sign detection algorithm proposed in this paper is compared with SSD, RetinaNet, FCOS, YOLOv3, YOLOv3-Tiny, YOLOv5-GhostNet, YOLOv5-MobileNet, and YOLOv5s on the TT-100K dataset The experimental results are shown in Table 2. It can be seen from Table 2 that the proposed SCB-YOLOv5 algorithm has little difference from YOLOv5s in terms of accuracy and recall. However, the mAP@50 of SCB-YOLOv5 is 0.2% less than that of YOLOv5s, and the inference speed is 20.8% faster than that of YOLOv5s. Compared with other algorithms, SCB-YOLOv5 has obvious advantages in speed and accuracy.

**Table 2:** Comparison of SCB-YOLOv5 detection algorithm with other algorithms.

Method	P	R	mAP@50	Inference time	FPS
**YOLOv3**	71.1	67.0	73.5	21.0ms	46.9
**YOLOv3-tiny**	79.3	64.8	72.7	7.9ms	108.7
**YOLOv5-Ghost**	64.1	57.2	60.7	9.5ms	92.6
**YOLOv5-MobileNet**	62.4	48.8	53.5	9.3ms	94.3
**SSD**	71.9	60.1	69.7	18.5ms	50.5
**RetinaNet**	72.4	50.6	62.4	31.0ms	30.9
**FCOS**	72.3	65.4	70.1	56.0ms	17.5
**YOLOv5s**	75.1	70.2	75.1	8.6ms	101.0
**ours**	78.8	69.4	74.9	6.9ms	122.0

Table 3 shows the comparison between SCB-YOLOv5 and other algorithms in terms of the number of parameters, amount of computation, and model size. Among them, SCB-YOLOv5 has the smallest model size, the least number of parameters, and the least amount of calculation. Compared with YOLOv5s, the number of parameters of SCB-YOLOv5 is reduced by 50.8%, the amount of calculation is reduced by 59.8%, and the model size is reduced by 48.8%.

**Table 3:** Comparison of the number of parameters and calculation of SCB-YOLOv5 detection algorithm with other algorithms.

Method	Parameters	Computational complexity (GFLOPs)	Model size
**YOLOv3**	61394M	156.6G	123.9MB
**YOLOv3-tiny**	8.85M	13.3G	17.6MB
**YOLOv5-Ghost**	2.98M	6.7G	6.5MB
**YOLOv5-MobileNet**	2.39M	4.6G	5.3MB
**SSD**	24.86M	62.4G	192.2MB
**RetinaNet**	36.15M	82.04	290.5MB
**FCOS**	31.84M	78.67G	256.2MB
**YOLOv5s**	6.27M	14.2G	13.1MB
**ours**	3.08M	5.7G	6.7MB

It can be seen from Tables 2 and 3 that SCB-YOLOv5 has obvious advantages over other mainstream detection algorithms in the number of model parameters, calculation, accuracy, and speed indicators.

**Fig 11:**
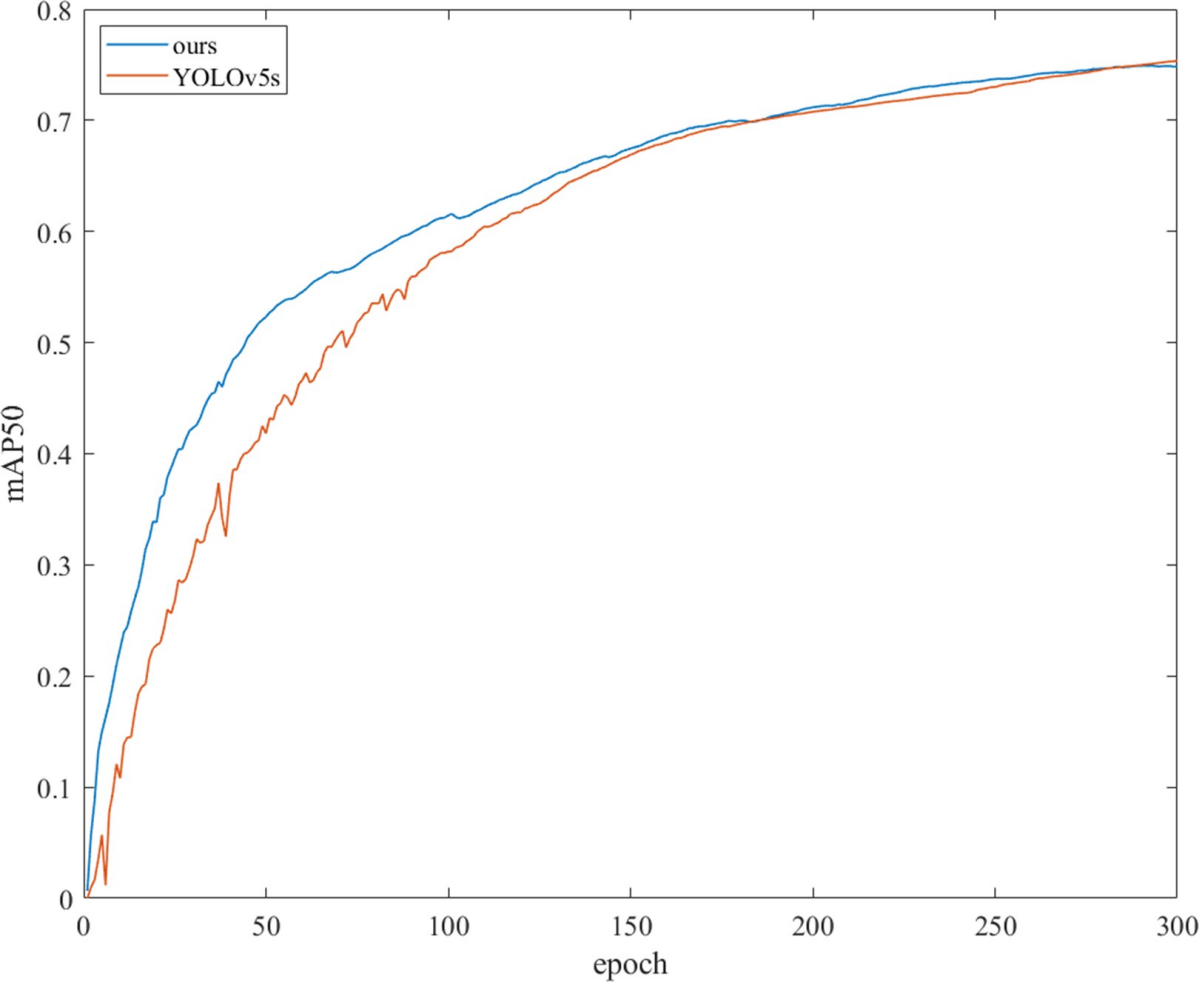
The comparison curves of mAP@50 between SCB-YOLOv5 and YOLOv5s. The abscissa is the number of training epochs, and the ordinate is mAP@50.

In the training process, the mAP@50 curves of SCB-YOLOv5 and YOLOv5s with epoch are shown in Fig 11. It can be seen from Fig 11 that SCB-YOLOv5 has a faster training convergence speed.

**Fig 12:**
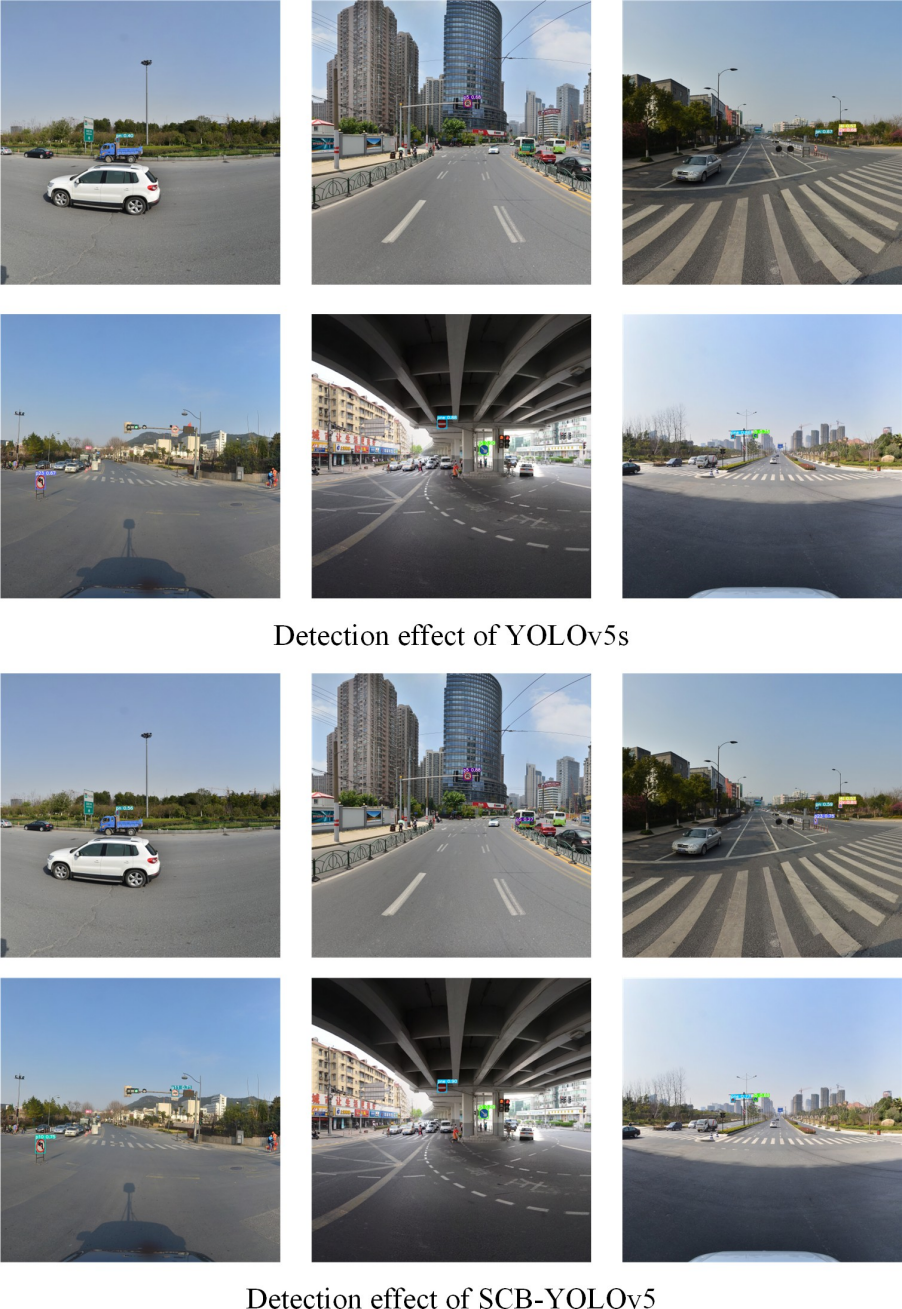
Comparison of detection effect between SCB-YOLOv5 and YOLOv5s.

Finally, in order to observe the comparison of the detection effect of SCB-YOLOv5 and YOLOv5s more intuitively, Fig 12 shows the comparison of the detection effect of SCB-YOLOv5 and YOLOv5s. As can be seen in Fig 12, SCB-YOLOv5s has higher detection confidence scores for traffic sign targets.

### 3.4 Ablation experiment

To verify the effectiveness of adding each improved structure to the proposed SCB-YOLOv5 detection algorithm, this paper conducted ablation experiments. The results of ablation experiments are shown in Table 4. As can be seen from Table 4, after replacing the backbone network with ShuffleNet v2, the size of the space occupied by the model, the number of parameters, and the amount of calculation are greatly reduced. Due to the simplification of the network, the mAP is reduced and the inference speed of the model is greatly improved. After adding the CA attention mechanism, the mAP is improved, but the speed is decreased. After adding BCS-FPN, the number of parameters of the model decreases, the mAP is reduced, and the inference speed is improved. Finally, compared with YOLOv5s, the proposed SCB-YOLOv5 model has the same accuracy, but the inference speed is greatly improved, and the number of parameters and calculations is greatly reduced.

**Table 4:** Comparison of algorithm results before and after adding different structures.

ShuffleNet+ SimSPPF	CA	C2f-SCConv	mAP	Inference time	FPS
√			73.8	7.5ms	113.6
√	√		74.4	7.7ms	111.1
√		√	73.9	6.7ms	125.0
√	√	√	74.9	6.9ms	122.0

### 3.5 Model deployment experiment

**Fig 13:**
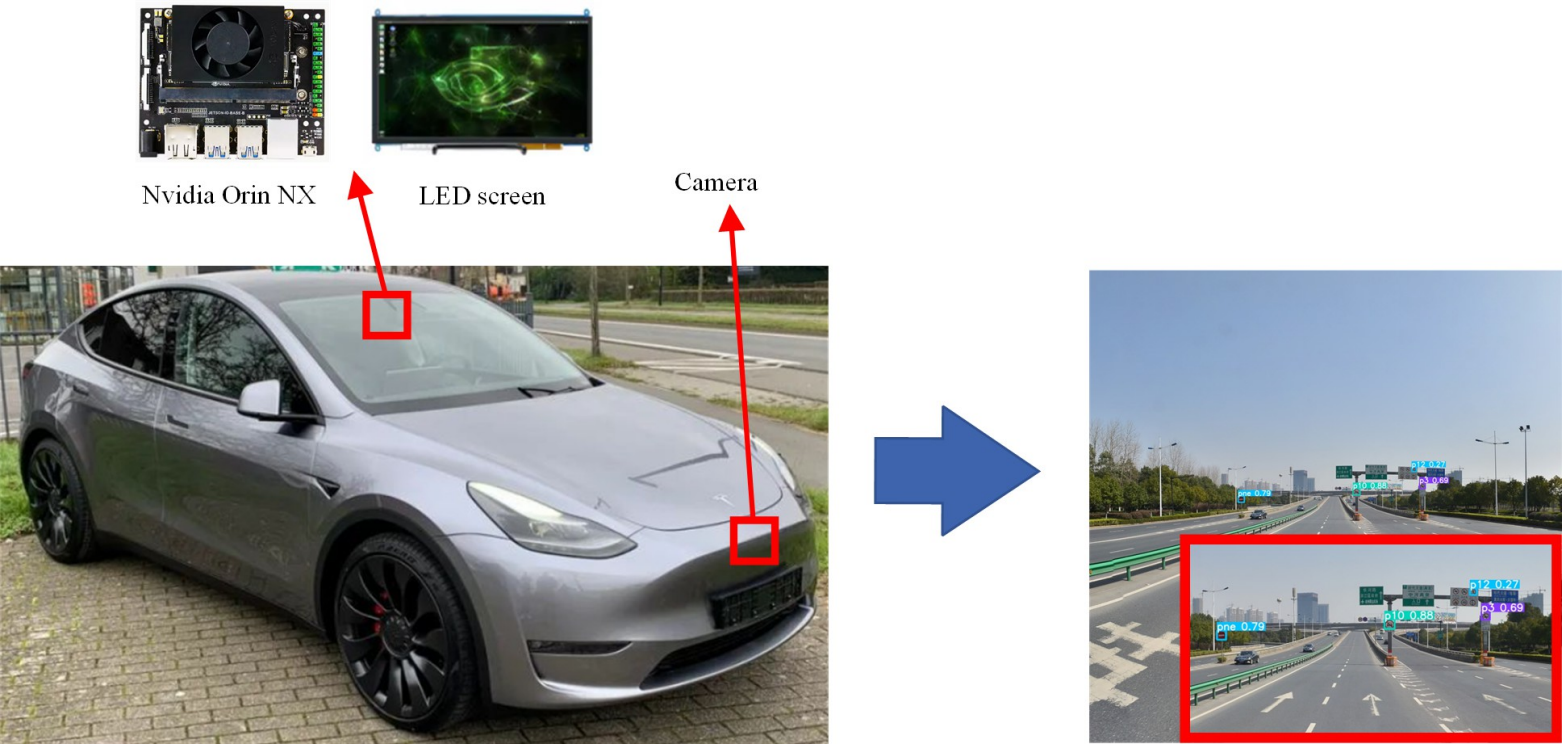
Schematic diagram of the traffic sign detection model deployment.

In order to verify the performance of the proposed SCB-YOLOv5 detection algorithm on embedded devices, this paper also conducted model deployment experiments on Nvidia Orin NX. The CPU of the Nvidia Orin NX is a 6-core NVIDIA CarmelARMv8.2 64-bit CPU. The GPU for Nvidia Orin NX is the NVIDIA Volta architecture with 384 NVIDIA CUDA cores and 48 Tensor cores. And it has 8GB of RAM. A schematic of the deployment on Nvidia Orin NX is shown in Fig 13.

In this paper, SCB-YOLOv5 is deployed with YOLOv5s, YOLOv3-tiny, YOLOv5-GhostNet, and YOLOv5-MobileNet v3 on Nvidia Orin NX, and the experimental results are shown in Table 5. It can be seen from Table 5 that the SCB-YOLOv5 detection algorithm proposed in this paper can perform real-time detection of traffic signs on embedded devices, and it is faster than other lightweight algorithms such as YOLOv5s, and the model size is also the smallest. In summary, the excellent performance of SCB-YOLOv5 on embedded devices is verified.

**Table 5:** Embedded end deployment experimental results.

Method	Inference time	Model size
**YOLOv3-tiny**	27ms	17.6MB
**YOLOv5-Ghost**	35ms	6.5MB
**YOLOv5-MobileNet**	37ms	5.3MB
**YOLOv5s**	31ms	13.1MB
**ours**	14ms	6.7MB

## IV. Conclusion

Aiming at the problems of road traffic sign detection, such as complex traffic sign target background, low saliency, large scale difference, large parameter amount of common target detection algorithm, and complex model, this paper proposes a lightweight traffic sign detection algorithm SCB-YOLOv5. Firstly, ShuffleNet v2 was used to replace the YOLOv5’s backbone network for extracting features, which greatly reduces the number of network parameters and improves the speed of network operation. And SimSPPF was used to replace SPPF, which improves the feature extraction ability of the backbone network. Then, the CA attention mechanism is added to the backbone network, which enhances the saliency of the object at the cost of a small computational cost. Finally, the BCS-FPN structure is designed to improve the feature fusion ability of multi-scale objects while reducing the amount of calculation. SCCBL is used as the convolution module for the BCS-FPN to reduce the amount of model calculation while ensuring the accuracy. The C2f-SCConv structure is designed to further reduce the number of network parameters and improve the detection speed. Moreover, the multi-scale feature fusion mechanism is introduced to improve the network feature fusion ability. In this paper, experiments are carried out on the TT-100K dataset. Experiments show that the mAP@50 of SCB-YOLOv5 reaches 74.9%, and the inference speed is 6.9ms, which is 20.8% higher than that of YOLOv5. This paper also conducts a deployment experiment on the embedded side. Experiments show that SCB-YOLOv5 can detect traffic signs in real-time on embedded devices. During the analysis of the data set, we found that the data set has fewer categories and the training effect is general. The next step of this paper is to first expand the data set to make the trained model generalize better. The second is to verify the detection ability of the algorithm for small-scale targets, and enhance the detection effect of the algorithm for small-scale targets. Finally, in the aspect of positive and negative sample matching and training strategy of the algorithm, we will use more appropriate optimization algorithms and matching algorithms [40, 41] to improve the efficiency of the model.

## Supporting information

S1 File Results for each epoch during model training. S1 File is the supporting information of Fig 11. Among them, results_for_YOLOv5.csv is the data of YOLOv5 during the training process, and results_for_SCB-YOLOV5.csv is the data of SCB-YOLOv5 during the training process. In the data, the first column is the number of training epoch, and the 2–4 columns are the changes in the value of each loss function during training. The 5–7 columns are the changes of precision, recall, and mAP@0.5. The 8–10 columns are the changes in the value of each loss function during validation process, and the last 3 columns are the change in learning rate.
(ZIP)

S2 File Public dataset sources have used in this paper. TT-100K dataset is used in this paper, and we selected traffic signs with more than 100 labels in the dataset as detection targets.
(DOCX)

S3 File SCB-YOLOv5 model configuration file and its core code. Among them, the core_code_for_SCB-YOLOv5_model_configuration_file.txt file is the core code of main structure in SCB-YOLOv5, and the SCB-YOLOv5.yaml file is the model parameter configuration file of SCB-YOLOv5.
(ZIP)
