# Supplementary material for: Improved lightweight YOLOv5 based on ShuffleNet and its application on traffic signs detection
Source: PLoS One. 2024 Sep 10;19(9):e0310269. doi: 10.1371/journal.pone.0310269 (PMC11386454; doi:10.1371/journal.pone.0310269)
Supplement: S2 File — TT-100K dataset is used in this paper, and we selected traffic signs with more than 100 labels in the dataset as detection targets. (DOCX) [file pone.0310269.s002.docx]

The TT-100K dataset is linked by <https://cg.cs.tsinghua.edu.cn/traffic-sign/>. The traffic signs specifically represented by the targets in the dataset are shown in the figure below.


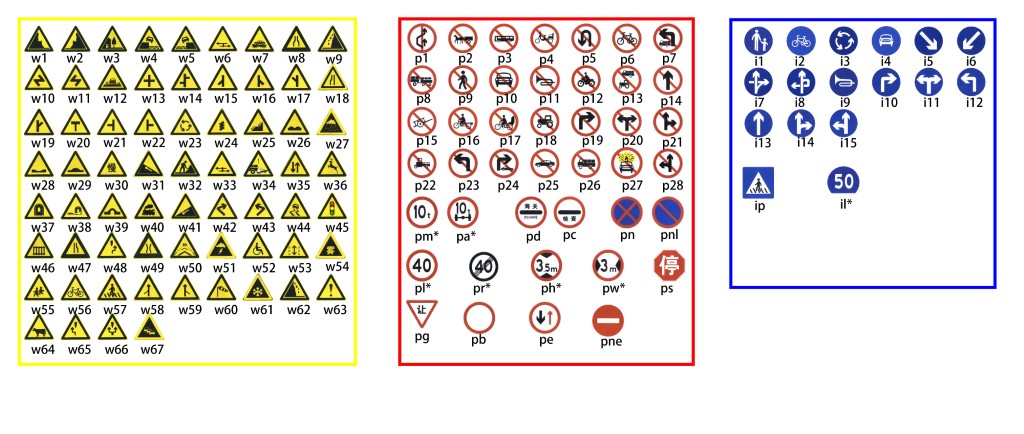


We selected traffic signs with more than 100 labels in the dataset as detection targets. After selection, a total of 35 classes are obtained as follows:

pne:2091;

pn:2963;

pl40:1356;

p5:393;

i2r:417;

pl50:1027;

pl60:820;

i5:1582;

w57:393;

pl80:865;

p12:181;

pl120:296;

pr40:200;

pl5:484;

w59:196;

pl30:596;

i4:733;

pm20:156;

ip:340;

pl100:664;

p23:281;

pm30:107;

pg:154;

p26:812;

w55:175;

p10:358;

p11:1535;

p13:353;

p19:122;

i4l:330;

ph4.5:186;

pl20:158;

p3:169;

pl70:149;

il80:294.
